# Febrile Temperature Augments Ring-stage *Plasmodium falciparum* Adhesion to Brain Endothelial Cells

**DOI:** 10.1093/infdis/jiaf474

**Published:** 2025-09-10

**Authors:** Fatou Joof, Ruoqian Hu, Karl B Seydel, Lauren M Cohee, Ying Zheng, Joseph D Smith

**Affiliations:** Center for Global Infectious Disease Research, Seattle Children's Research Institute, Seattle, Washington, USA; Department of Bioengineering, University of Washington, Seattle, Washington, USA; Blantyre Malaria Project, Kamuzu University of Health Sciences, Blantyre, Malawi; Department of Osteopathic Medical Specialties, College of Osteopathic Medicine, Michigan State University, East Lansing, Michigan, USA; Center for Vaccine Development and Global Health, University of Maryland School of Medicine, Baltimore, Maryland, USA; Department of Clinical Sciences, Liverpool School of Tropical Medicine, Liverpool, United Kingdom; Department of Bioengineering, University of Washington, Seattle, Washington, USA; Center for Global Infectious Disease Research, Seattle Children's Research Institute, Seattle, Washington, USA; Department of Pediatrics, School of Medicine, University of Washington, Seattle, Washington, USA

**Keywords:** cerebral malaria, cytoadhesion, febrile temperature, 3D brain microvessel, ring stage

## Abstract

Sequestration of *Plasmodium falciparum*–infected erythrocytes (IE) in the microvasculature is a major virulence determinant. While the sequestration of mature stage parasites (trophozoite and schizonts) to vascular endothelium is well established, the conditions that promote ring-stage IE sequestration are less understood. Here, we observed in ring-stage parasites that febrile exposure increased transcript levels of several exported parasite genes involved in the trafficking of the *P. falciparum* erythrocyte membrane protein 1 (PfEMP1) ligand responsible for adherence to the endothelium of blood vessels. Furthermore, it accelerated PfEMP1 surface display in ring-stage IEs, leading to a 2-fold increase in their binding in a perfusable 3D human brain microvessel model. Additionally, we observed that parasite exposure enhances the binding of uninfected erythrocytes in 3D brain microvessels. These findings suggest a complex interplay between fever and parasite biomass in the pathogenesis of cerebral malaria.

## Teaser

Cerebral malaria is characterized by vascular obstruction by infected erythrocytes. This study shows that febrile temperature enhances ring-stage *Plasmodium falciparum* adhesion to brain endothelium by accelerating exportome transcription and *P. falciparum* erythrocyte membrane protein 1 surface display, suggesting that fever promotes earlier parasite–vascular interactions.

Cerebral malaria is a severe neurological complication of *Plasmodium falciparum* infection, characterized by coma and sequestration of infected erythrocytes (IE) in the brain microcirculation [1–3]. While sequestration is primarily mediated by mature stage trophozoite and schizont-IEs [1, 2, 4, 5], blood transcriptional profiling of infected people indicate that immature ring-stage IEs disappear more rapidly from blood circulation in symptomatic infections [6] and severe infections [7]. From postmortem brain autopsies, mature trophozoite and schizont IEs predominant in the cerebral microvessels [1, 2, 4, 5], but ring-stage IEs can also be detected [8], and overall microvascular congestion is due to both infected and uninfected red blood cells [9]. Collectively, these findings indicate that sequestration may not be restricted to mature parasite stages, but the conditions that influence ring-stage IE adherence to vascular endothelium remain poorly characterized.

During the blood stage of infection, *P. falciparum* parasites export a suite of proteins into the host erythrocyte, altering its mechanical and adhesive properties [10–12]. Central among these is *P. falciparum* erythrocyte membrane protein 1 (PfEMP1), a clonally variant adhesion ligand encoded by the *var* gene family and presented on knob-like protrusions in the erythrocyte membrane [13–16]. Cerebral malaria and other forms of severe malaria are associated with a subset of PfEMP1 variants that engage endothelial protein C receptor (EPCR) [17–20]. While the roles of most exported proteins remain poorly defined, key effectors such as the PfEMP1 trafficking proteins (PTPs) [21, 22], Dnaj heat shock proteins [23], and FIKK kinases [24, 25] mediate PfEMP1 delivery to knob structures and facilitate cytoadhesion. Other exported factors enhance IE rigidity and remodel the erythrocyte cytoskeleton to support sequestration [11, 21].

Cyclical fever, a hallmark of symptomatic malaria infections, is triggered by the host inflammatory response to the rupture of schizont-IEs [26]. Previous in vitro studies have shown that while extended febrile conditions can inhibit the growth of mature-stage IEs [27, 28], shorter exposures of 2 to 6 hours increase transcript levels of several parasite exported proteins [29] and accelerates PfEMP1 surface display in ring-stage IEs, enhancing their adherence to CD36 and intercellular adhesion molecule 1 [30]. These findings highlight a potential role of fever in promoting the cytoadherence of younger developmental parasite stages, but its impact has not been addressed in a brain endothelial model. To address mechanisms linking fever and parasite sequestration to the development of severe malaria, we used an EPCR-binding parasite line and a flow-based 3D human brain microvessel model [31] to study how febrile temperature and parasite density may jointly influence the interactions of ring-stage IEs and uninfected red blood cells with human brain endothelial cells.

## METHODS

### Human Plasma Samples

Plasma was obtained from blood samples that were selected from a longitudinal cohort study in Southern Malawi [32].

### Ethical Approval and Consent to Participate

This study was approved by the Institution Review Boards of the Malawi National Health Science Research Committee, Michigan State University, and University of Maryland.

### *P. falciparum* Culture

This study used *P. falciparum* lab line IT4var19 that was selected on human brain endothelial cells [33] and FCR3-CSA that was selected on chondroitin sulfate A [34]. Parasites were maintained in human red blood cells (O+) and 10% pooled human A+ serum-rich RPMI 1640 medium at 37°C. Synchronized asexual *P. falciparum* cultures were obtained by treatment with 5% D-Sorbitol (Sigma-Aldrich) and by passing the IT4var19 parasite culture through a magnetic columns (MACS) separator (LD columns, Miltenyi Biotec) for trophozoite enrichment.

### Heat Shock Experiments

Synchronized early ring-stage IEs 0–6 hours post invasion (hpi) at 5% to 18% parasitemia were maintained at 40°C and a control parasite culture at 37°C for 4 hours. Subsequently, both flasks were incubated at 37°C for another 4 hours. At the end of the 8 hours, IEs (8–14 hpi) were used for downstream analysis: transcriptomic profiling using nCounter NanoString, surface antigen reactivity using flow cytometry and perfusion into 3D brain microvessel. For flow cytometer analysis, continuous parasites culture was maintained at 37°C for up to an additional 16 hours. For uninfected erythrocytes (UE) heat shock, the same procedure was used.

### nCounter Analysis

For transcription analysis, we used a custom nCounter code set (NanoString) of exported malaria genes. Our custom code set consists of 50 gene targets, including 3 housekeeping genes (glyceraldehyde 3-phosphate dehydrogenase, GAPDH; seryl-tRNA synthetase, STS; arginyl-tRNA synthetase, ATS) and the IT4var19 and var2CSA genes expressed by the respective parasite lines (Supplementary Table 1). We used whole parasite cell lysate for sample input. In brief, the cycle before heat treatment, magnetic activated cell sorting (MACS) enrichment was used to isolate mature *P. falciparum*-IEs. The newly invaded ring-stage parasite culture (0–6 hpi) was treated with sorbitol to kill mature *P. falciparum* blood stages, adjusted to 5% parasitemia, and divided into flasks that were incubated for 4 hours at 37°C or 40°C, followed by 4 hours at 37°C for both. For nCounter analysis, 100 000 intact parasites were obtained by lysing the red blood cell membrane with 0.4% saponin and hemocytometer count. A parasite cell lysate was then prepared by lysis with buffer RLT (Qiagen) with 1:100 of ß-mercaptoethanol (GIBCO) and frozen at −80°C until submission for nCounter analysis. The STS transcript was used for normalization between the 2 temperature conditions.

### Flow Cytometry

Surface reactivity of IE following heat shock was assessed by flow cytometer with a pool of 3 Malawian plasma at 4 timepoints: 0 hours—at the end of heat shock treatment (parasites, 8–14 hpi), + 2 hours (parasites, 10–16 hpi), + 4 hours (parasites, 12–18 hpi), and +16 hours (parasites, 24–30 hpi). IEs nuclei were stained with SYBR green DNA stain (Thermo Fisher Scientific, 1:5000) and incubated with 1:10 dilution of pooled Malawian plasma samples for an hour at 37°C. Cells were then washed and labeled with goat anti-human IgG Alexa Fluor 647 (Invitrogen, 1:200 dilution) in 1 × phosphate-buffered saline (PBS) containing 0.5% BSA for 30 minutes at room temperature. Flow cytometry was conducted on an LSR II flow cytometer (BD Biosciences). The mean fluorescence intensity (MFI) of Alexa Fluor 647 was obtained from 10 000 SYBR green-positive events. Data was analyzed using FlowJo (10.8.1 TreeStar Inc).

### Human Brain Endothelial Cell Culture

Primary human brain microvascular endothelial cells (HBMECs; Cell Systems, ACBRI 376, passage 3) underwent expansion and culture, as described previously [31]. One million HBMECs were plated in a T75 tissue culture flask (Corning) coated with Attachment Factor (Cell Systems) and maintained at 37°C with 5% CO_2_. Growth medium consisted of EGM-2 Basal Medium (Lonza) supplemented with EGM-2 MV Microvascular Endothelial Cell Growth Medium SingleQuots (Lonza) at 37°C with 5% CO_2_. HBMEC cells between passage 3 and 6 were used for 3D microvessel assembly.

### Brain Microvessel Fabrication

Three-dimensional brain microvessels were fabricated in a 13 × 13 network geometry using 7.5 mg/mL type I collagen, following our established protocol [31]. Briefly, a micropatterned top collagen piece and a flat collagen piece were assembled to form perfusable microchannels connected to inlet and outlet media reservoirs. Each microvessel was then endothelialized by seeding 200 000 HBMECs via direct perfusion-based seeding from both inlet and outlet reservoirs. Microvessels were cultured under gravity-driven perfusion in EGM2-MV growth medium, with media refreshed twice daily for 3–5 days post-fabrication to establish a confluent endothelial lumen.

### Perfusion of 3D Brain Microvessels

To assess the effect of febrile temperature on red blood cell binding in 3D brain microvessels, synchronized ring-stage IT4var19-IEs (8–14 hpi, 5–18% parasitemia) were either heat shock treated (40°C for 4 hours, followed by 37°C for 4 hours) or maintained at 37°C throughout. Heat-shocked IEs were labeled with PKH26 red (Sigma) and control IEs with PKH67 green (Sigma), per manufacturer instructions. Membrane labelled erythrocytes were counted with a hematocytometer and mixed to 5 × 10^6^ cells/mL in a 1:1 ratio. To perfuse the microvessel, 150 µL of the IE mixture was introduced to the inlet and 50 µL of EGM-2MV medium to the outlet for 15 minutes. Microvessels were then washed twice (15 minutes each) with EGM-2MV media, fixed in 3.7% paraformaldehyde/0.008% glutaraldehyde in PBS, and stained with Hoechst (Thermofisher).

### Parasite Binding Quantification

Fixed and stained microvessels were imaged as previously described [35]. Briefly, 4-channel z-stack images spanning the lumen surface were acquired with 5–10 µm z-step sizes and then stitched together to reconstruct the entire 13 × 13 microvessel network. IE binding was quantified by counting double-positive erythrocytes with membrane-label (green or red) and parasite nuclei as previously described [35], while UE binding was determined by membrane-label positive but nuclei-negative cells. Phase contrast images were used to verify erythrocyte morphology for all cell counts.

### Statistical Analysis

Statistical analyses were conducted using GraphPad Prism software (version 10.0.3, Graphpad Software Inc.). Statistical significance of differences between bound IEs populations (40°C vs 37°C) was assessed using the unpaired two-tailed *t* test with *P* < .05 considered statistically significant.

## RESULTS

### Elevated *P. falciparum* Exportome Transcripts in Ring-stage Parasites Exposed to Febrile Temperature

**Figure 1.**
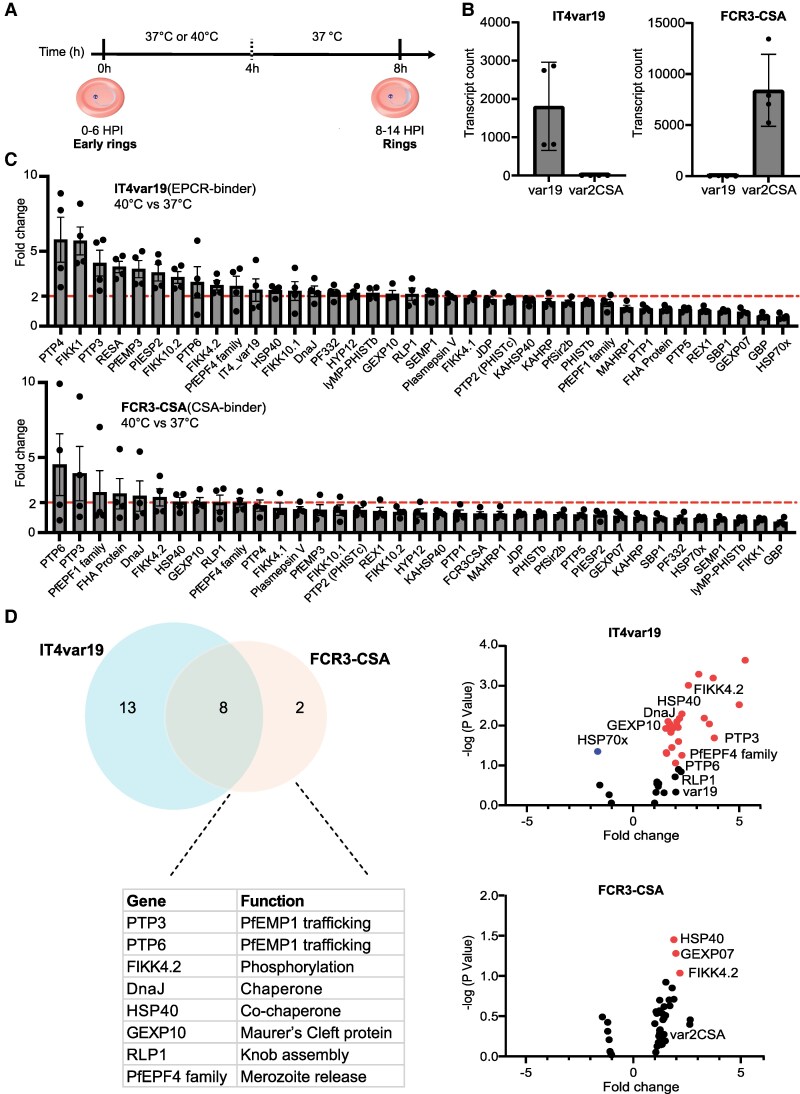
Transcriptional profiling of *Plasmodium falciparum* exportome in ring-stage parasites in response to febrile exposure. *A,* Heat shock experiment timeline. *B,* Bar charts show *var* transcript counts in the IT4var19 and FCR3CSA parasite lines using nCounter analysis (mean ± SEM) (n = 4). *C,* Differential gene expression analysis of the *P. falciparum* exportome in response to febrile temperature. The febrile-induced fold change in gene transcript levels between parasite lines exposed to 40°C compared to 37°C was normalized to the housekeeping gene seryl-tRNA synthetase (STS). A 2-fold change threshold is indicated by a dotted red line (n = 4, independent biological replicates). *D,* Venn diagram showing the number of febrile-elevated genes (≥2 fold) and volcano plot showing differentially expressed genes following febrile treatment of the IT4var19 and FCR3CSA parasite lines. The blue and red dots represent significantly differentially expressed (down/up-regulated genes) under febrile condition (unpaired *t* test, *P* < .05). Abbreviation: HPI, h post-infection.

Fever is a common symptom of cerebral malaria and malaria during pregnancy and distinct parasite binding variants are involved in each disease syndrome. We first assessed the impact of febrile conditions on ring-stage *P. falciparum* exportome gene expression by using the EPCR-binding IT4var19 parasite line to represent pediatric cerebral malaria variants [17, 20, 33], and the FCR3CSA parasite line for placental malaria [34]. To simulate a febrile episode triggered by the rupture of IEs, early ring-stage parasites (0–6 hpi) were maintained for 4 hours at 40°C versus 37°C and then returned to 37°C for 4 hours (Figure 1*A*).

Transcriptomics analysis was performed using a custom nCounter code panel specifically designed for exported *P. falciparum* genes (Supplementary Table 1) using 100 000 parasites for all experiments (Supplementary Figure 1). As expected, the 2 *var* genes were differentially expressed by the parasite lines: *var19* transcripts predominated in IT4var19 (1806 ± 575 *var19*) with minimal *var2CSA* (1.42 ± 0.5), whereas FCR3CSA showed the opposite pattern (8573 ± 1764 *var2CSA*; 14 ± 12 *var19* transcripts; mean ± SEM; Figure 1*B*). For the remaining genes in our code set, 9 genes were eliminated due to low transcript counts (<70 transcript counts). *RESA* was not detected in the FCR3CSA parasite line due to a known deletion event that occurred during parasite cultivation [36], leaving 37 exported genes for direct comparison (Figure 1*C*). We defined a 2-fold change of normalized transcript counts between 40°C versus 37°C, as differentially expressed. By this criterion, we observed 21 differentially expressed transcripts with the IT4var19 parasite line and 10 for the FCR3CSA parasite line (Figure 1*C*), of which 8 were shared febrile-responsive transcripts (Figure 1*D*).

Among the shared febrile-increased transcripts were the PTP gene group (PTP3 and PTP6) and a FIKK kinase (FIKK 4.2) (Figure 1*C* and 1*D*). In the IT4var19 parasite line, PTP4 exhibited the highest fold-increase, alongside several additional FIKK family members. The PTP genes facilitate PfEMP1 trafficking to the erythrocyte membrane [21, 22], whereas FIKK kinases are a family of parasite serine threonine kinase that are exported to the red blood cell and are known to phosphorylate host red blood cell and exported parasite proteins [24, 37]. Several genes implicated in chaperone functions, PfEMP1 trafficking, and/or heat shock response were elevated in response to 40°C in both parasite lines (Figure 1*C*), including *HSP40* [23, 38] and *GEXP10* [39]. *RESA*, which encodes a protein that binds to spectrin ensuring erythrocyte rigidity and may protect against thermal damage [40], was among the most highly upregulated transcripts in the IT4var19 parasite line (Figure 1*C*). Taken together, these data show that febrile temperature increased transcript levels of several exported proteins involved in PfEMP1 trafficking and surface display.

### Febrile Conditions Accelerate PfEMP1 Surface Display in Ring-stage *P. falciparum*-IEs

**Figure 2.**
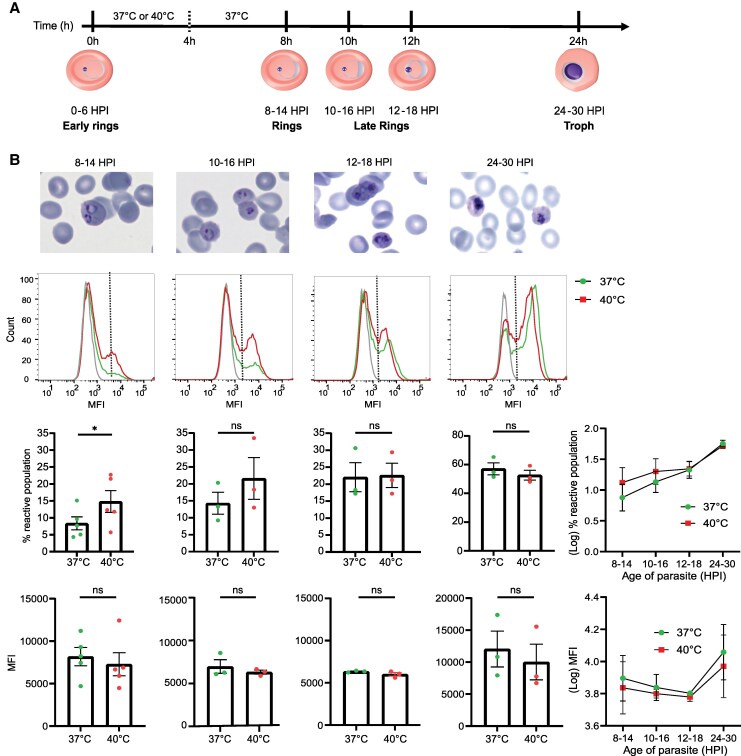
Febrile exposure accelerates the surface reactivity of ring-stage *Plasmodium falciparum*-IEs by Malawian plasma. *A,* Heat shock experiment timeline. *B,* Top: Representative Giemsa-stained smears of *P. falciparum*-IEs at different time points. Second row: Representative flow cytometry of Malawian plasma reactivity on *P. falciparum*-IEs exposed to 40°C compared to the 37°C control. Grey curve represents negative control (US plasma) surface reactivity. Vertical dotted line indicates cutoff for surface reactive population. Third row: Bar charts show percentage surface reactive population of *P. falciparum*-IEs (n = 3 to 5, independent biological replicates) (mean ± SEM; *P < .05, unpaired *t* test). The line plot shows different timepoints over parasite maturation (mean ± SEM). Fourth row: Bar charts show the MFI values of surface reactivity in *P. falciparum*-IEs (n = 3 to 5, independent biological replicates). The line plot shows different timepoints over parasite maturation (mean ± SEM). Abbreviations: ns, not significant; MFI, mean fluorescence intensity.

Febrile temperature is known to accelerate PfEMP1 surface display in ring-stage *P. falciparum*-IEs [30], but its effect has not been studied for the EPCR-binding subset of PfEMP1 variants, which are common in cerebral malaria infections [17–20]. To address this, we performed flow cytometry with a pool of Malawian plasma selected for high reactivity to the IT4var19 PfEMP1 variant [35] at different timepoints following febrile treatment (Figure 2*A*). At 8–14 hpi, ring-stage parasites exposed to 40°C showed a significant increase in surface reactive cells (14.8% ± 3.5) compared with 37°C (8.3% ± 2.1, *P* < .05). However, this difference diminished as the parasites matures: 10–16 hpi (40°C, 21.6% ± 5.3; 37°C, 14.3% ± 2.2), 12–18 hpi (40°C, 22.6% ± 3.1; 37°C, 22.3% ± 3.5), 24–30 hpi (40°C, 52.6% ± 2.5; 37°C, 57.1% ± 3.2) (Figure 2*B*). On the other hand, the MFI of antibody surface reactivity increased between ring-stage parasites (8–18 hpi) and trophozoite-stage parasites (24–30 hpi) (Figure 2*B*). However, there was no significant difference between 37°C and 40°C conditions at any time points: 8–14 hpi (40°C, 7280 ± 1528; 37°C, 8180 ± 1200), 10–16 hpi (40°C, 6315 ± 185; 37°C, 6973 ± 732), 12–18 hpi (40°C, 6017 ± 167; 37°C, 6297 ± 105), and 24–30 hpi (40°C, 10 005 ± 2409; 37°C, 12 041 ± 2421). Our data align with previous findings [30] that febrile-induced increases in PfEMP1 display are mainly restricted to ring-stage parasites.

### Febrile Treatment Induces a 2-Fold Increase in Ring-stage *P. falciparum*-IE Cytoadhesion in a Human 3D Brain Microvessel Model

**Figure 3.**
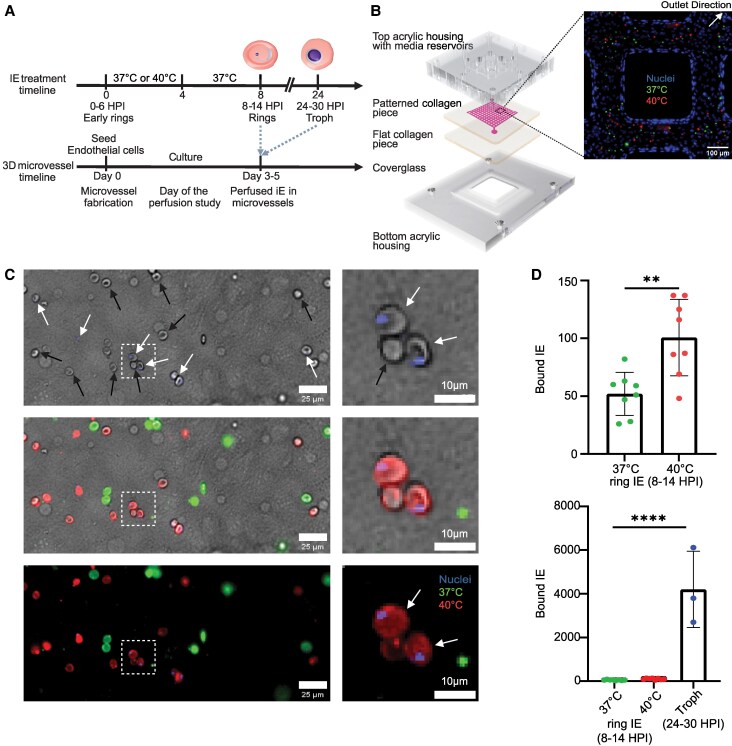
Febrile temperature exposure augments ring-stage parasite binding in 3D brain microvessels. *A,* Timelines of ring-stage parasite heat shock and perfusion in 3D brain microvessels. For a stage-dependent binding comparison, trophozoites (24–30 HPI) maintained at 37°C were perfused. *B,* Schematic of 3D brain microvessel device fabricated with a 13-by-13 network within a collagen hydrogel. Zoom-in image shows adherent red blood cells: 40°C (red fluorescent membrane stain) and 37°C control (green fluorescent membrane stain). Nuclei staining depicts microvessel lumen lined with endothelial cells. *C,* Representative bright-field image of a microvessel surface with multiple bound infected or uninfected red blood cells. Ring-stage IEs (white arrows) are double positive for Hoechst nucleic acid stain and red (40°C) or green fluorescent (37°C) membrane stain. Parasite-exposed UEs (black arrows) lack blue nuclei staining and are red (40°C) or green (37°C) fluorescent membrane stained. Right: Zoomed-in box inset shows a cluster of two infected cells (white arrow) and one uninfected cell (black arrow) adhering to the brain endothelial cells. *D,* Quantification of IE binding to endothelial cells in 3D human brain microvessels. Top: Bar chart shows the total bound counts of febrile-exposed (40°C) and control (37°C) ring-stage IEs (8–14 HPI) (mean ± SEM; **P < .01, unpaired *t* test). Bottom: Bar graph shows the total number of bound trophozoites as compared to ring-stage IE binding (mean ± SEM; ****P < .0001, unpaired *t* test). n = 8 independent 3D brain microvessels for ring-stage IEs (*C* and *D*) and n = 3 independent 3D brain microvessels for trophozoite-stage IEs (*D*). Abbreviations: 3D, 3 dimensional; HPI, hours post-infection; IE, infected erythrocytes; UE, uninfected erythrocytes; troph, trophozoites.

In fatal CM cases, trophozoites and schizonts predominate in the brain microvasculature, but immature ring-stage IEs can also accumulate [8]. The mechanism of how these early developmental stages interact with brain endothelial cells remains poorly understood. To investigate whether febrile exposure can increase the adherence of ring-stage IEs to human brain endothelial cells, we used a perfusable 3D brain microvessel model (internal diameter ∼100 µm), which we have previously applied to study the binding of trophozoite-stage IEs [31]. For this analysis, we co-perfused microvessels with an equal number of ring-stage IT4var19 IEs (8–14 hpi), cultured at 37°C (green fluorescent membrane stain) or exposed to 40°C (red fluorescent membrane stain) (Figure 3*A and B*). After perfusing microvessels for 15 minutes and washing unbound cells, we quantified the number of red and green IEs bound to microvessels, by using Hoechst nucleic acid stain to visualize parasitized cells (Figure 3*C*). We observed a nearly 2-fold increase in the number of bound ring-stage *P. falciparum*-IEs when parasites were exposed to febrile temperature (40°C, 100 ± 16.5; 37°C, 52 ± 9 [*P* < .01]) (Figure 3*D*). The binding levels of febrile-exposed, ring-stage IEs was approximately 40-fold lower than trophozoite-stage *P. falciparum*-IEs (24–30 hpi) that were never exposed to febrile conditions (4204 ± 856; *P* < .0001; Figure 3*D*). These findings indicate that mature-stage *P. falciparum*-IEs have substantially higher binding affinity to brain endothelial cells than ring-stage parasites and that febrile exposure augmented binding of ring-stage IEs.

### Cultivation of *P. falciparum* at High Parasitemia Increases Binding of Uninfected Red Blood Cells in 3D Brain Microvessels

**Figure 4.**
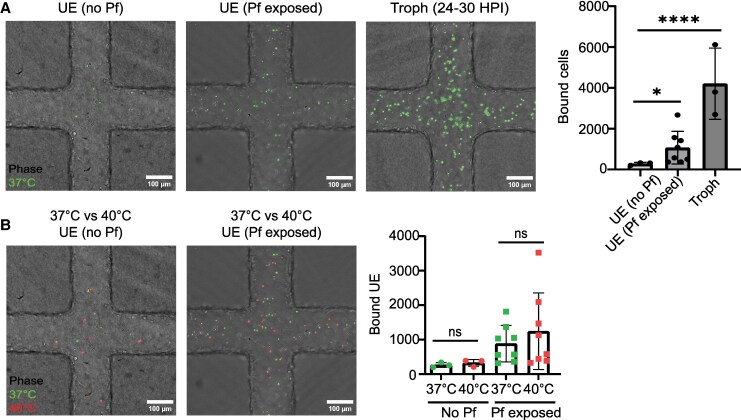
Cultivation of *Plasmodium falciparum* increased binding of uninfected red blood cells in 3D brain microvessels. *A,* Top: Representative bright-field images of non-parasitized green fluorescent labeled UE, either no parasite exposure (no Pf) or parasite-exposed (Pf exposed), and trophozoites binding in 3D human brain microvessels. Right: Bar chart of bound UEs (n = 8) and trophozoites (n = 3, same experiments as depicted in Figure 3). Mean ± SEM; *P < .05, ****P < .0001, unpaired *t* test. *B,* Effect of febrile exposure on binding of UE in 3D brain microvessels. Left: Representative bright-field images of green fluorescent labeled (37°C) and red fluorescent labeled (40°C) UEs binding, with no parasite exposure (no Pf) or parasite-exposed (Pf exposed). Right: Bar chart shows UEs binding in 3D microvessel when cultured in the absence of parasites (n = 3) and in the presence of parasites (n = 8, same experiments as depicted in Figure 3). Mean ± SEM; unpaired *t* test. Abbreviations: UE (no Pf), uninfected erythrocytes (human red blood cells O + sample) cultured alone; UE (Pf exposed), uninfected erythrocytes from a 5% to 18% parasitemia ring-stage IE culture; UEs, uninfected erythrocytes.

In severe falciparum malaria infections, uninfected red blood cells become less deformable [41, 42]. It has been postulated that altered red blood cells may hinder microcirculatory flow and contribute to organ damage in falciparum malaria, but this phenomenon has not been studied in microvessels. Due to the challenges of enrichment of ring-stage IEs from uninfected cells, we perfused microvessels with ring-stage IEs between 5% and 18% parasitemia (ratio of uninfected to infected cells: ≥ 5-fold). Unexpectedly, we observed UEs binding to the microvessels (Figure 3*C*). Conversely, there was limited binding of UEs not exposed to parasites (282 ± 19.2) compared with those cultured with parasites (1068 ± 141, *P* < .05) (Figure 4*A*). Since both fever and high parasite biomass are common in severe malaria, we then evaluated whether febrile condition alone may also influence the binding of UEs in brain microvessels. We did not observe a heat-induced difference in binding of parasite-free UEs (40°C, 327 ± 39 vs 37°C, 263 ± 35) or for parasite exposed UEs (40°C, 1243 ± 555 vs 37°C, 883 ± 256) (Figure 4*B*). Taken together, this analysis suggests that high parasitemia, but not febrile exposure, drive UE adhesion in our 3D brain microvessel model.

## DISCUSSION

The PfEMP1-mediated cytoadherence of mature-stage *P. falciparum*-IEs to host receptors like EPCR in the brain microvasculature contributes to the development of cerebral malaria [17–20]. However, recent findings indicate that ring-stage IEs may sequester from blood circulation in symptomatic or severe malaria infections [6, 7] and ring-IEs can bind to endothelial cells from lung, brain and synctiotrophoblast [8, 43], challenging the assumption that only mature stage parasites are capable of sequestering in the brain. Fever, a prominent symptom of severe malaria, has been proposed to influence the dynamics of parasite sequestration [30], although the extent and mechanisms of this influence remain poorly understood. Here, using a perfusable 3D human brain microvessel model, we show that ring-stage IEs adhere minimally to brain endothelium at normothermia and febrile temperature enhances binding.

Our study found that febrile exposure increased exportome transcripts and accelerated PfEMP1 surface display in ring-stage IEs. In alignment with previous findings [30], this effect waned with parasite maturation, suggesting a transient febrile adaptation specific to the ring-stage parasites. Notably, several transcripts associated with the cytoadhesion machinery were elevated in an EPCR-binding parasite line, a trait linked to severe malaria [17–20]. This included PTP3, PTP4, and PTP6 involved in PfEMP1 trafficking from the Maurer's clefts to the erythrocyte membrane [21, 22], as well as multiple FIKKs [37, 44], a family of kinases that are known to phosphorylate host and parasite proteins and modulate red blood cell rigidity [25] and parasite cytoadherence [24, 37]. FIKK4.2, an exported kinase involved in knob assembly and cytoadhesion that phosphorylates erythrocyte spectrin and the parasite knob associated histidine rich protein [24, 25] was upregulated in both parasite lines, pointing to potential febrile-induced alterations in parasite kinase regulators of cytoadhesion. We also detected upregulation of RESA in the IT4var19 parasite line, a protein implicated in RBC stabilization during heat exposure [40]. Our data extend previous work observing changes in a FIKK kinase and other exported gene products [29] by specifically linking fever-induced exportome modulation to parasite-brain endothelial interactions. Future work should explore whether fever accelerates maturation of ring-stage parasites by examining other gene expression markers of trophozoite transition.

Compared to mature stage parasites, ring-stage IEs have 40- to 80-fold lower interaction with brain endothelial cells. The fever-induced doubling of ring-stage IE cytoadherence could potentially serve as an early foothold for vascular adhesion and confer a competitive growth advantage by allowing parasites to evade splenic clearance mechanisms, contributing to decreased parasite circulation times and higher parasite burdens in severe infections [7]. We did not assess febrile responses in trophozoite parasite stages. However, Udomsangpetch et al. [30] found that the increased adhesiveness of febrile exposed ring-stage IEs to immobilized CD36 and ICAM-1 waned as parasites matured to trophozoites. Other investigators have exposed trophozoite stage parasites to brief febrile temperatures and showed slight increases in adhesion to chondroitin sulfate A and CD36 at lower flow rates [45, 46]. Our study suggests that fever, a common symptom of infection, may inadvertently enable more rapid sequestration of ring-stage parasites to vascular endothelial cells and lead to higher parasite sequestration in the brain.

Unexpectedly, we also observed that parasite exposure increased UE binding in 3D brain microvessels. This effect was parasite-dependent but not influenced by febrile conditions. While previous studies have shown that severe falciparum malaria alters the deformability of uninfected red blood cells [41, 42], there is limited understanding about how it may influence red blood cell movement and interactions within the small blood vessels. The large diameters (∼100 µm) of our microvessels suggest that the observed UE retention is not simply due to mechanical trapping. Aside from direct endothelial binding, an alternative mechanism for UE binding may involve rosetting interactions between UEs and infected cells [47, 48], but many attached UEs were not adjacent to infected cells. Further studies are needed to fully understand the mechanisms by which parasite exposure increases the adhesion of uninfected red blood cells in 3D brain microvessels.

A limitation of our study is the microvessels have larger internal diameters that are not suitable for studying capillary blockages. Because the flow dynamics within microvasculature are strongly influenced by vessel diameter, the conclusions drawn here may not be fully translatable to smaller microvessels. Future work in narrower microvessel models that are designed to study the biophysical properties of erythrocyte deformability flowing through capillary-sized transitions [49] may allow additional mechanistic insight into how factors like fever and parasite density impact microvascular congestion in malaria. In addition, this study investigated resting microvessels, but fever and inflammation often co-exist in malaria infection. We previously found that mature-stage IEs were sensitive to changes in receptor expression on tumor necrosis factor-activated endothelial cells [31]. Future work should consider the potential complex interplay of fever and inflammation on both parasite and endothelial cells that may influence the extent of parasite cytoadherence.

In summary, our findings provide evidence for a febrile temperature-dependent mechanism of interaction between ring-stage IEs and brain endothelial cells, which could inadvertently facilitate parasite sequestration and contribute to disease severity.

## Supplementary Material

jiaf474_Supplementary_Data

## Supplementary Data

Supplementary materials are available at *The Journal of Infectious Diseases* online (http://jid.oxfordjournals.org/). Supplementary materials consist of data provided by the author that are published to benefit the reader. The posted materials are not copyedited. The contents of all supplementary data are the sole responsibility of the authors. Questions or messages regarding errors should be addressed to the author.
