# Supplementary material for: Febrile Temperature Augments Ring-stage Plasmodium falciparum Adhesion to Brain Endothelial Cells
Source: J Infect Dis. 2025 Sep 10;233(5):e1215–25. doi: 10.1093/infdis/jiaf474 (PMC13175607; doi:10.1093/infdis/jiaf474)
Supplement: jiaf474_Supplementary_Data [file jiaf474_supplementary_data.zip › Supplemental_Figure1.docx]

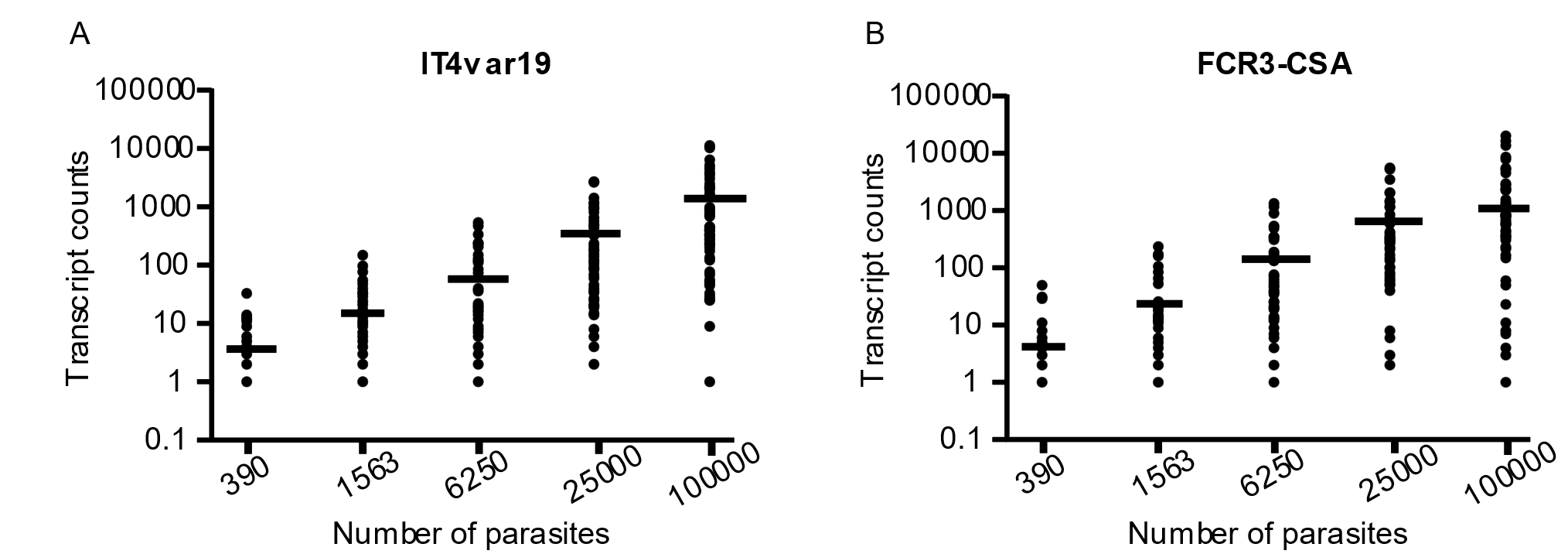


**Figure S1.** Serial dilution of parasites to determine transcript counts using nCounter analysis. Strip plot shows the relationship between the number of whole parasite cells used for lysate preparation and the corresponding transcript counts (median) for the IT4var19 and FCR3CSA parasite lines.

**Figure S1 ALT TEXT:** Graphs and data on the relationship between transcriptional levels and the input number of parasites.
