# Supplementary material for: Febrile Temperature Augments Ring-stage Plasmodium falciparum Adhesion to Brain Endothelial Cells
Source: J Infect Dis. 2025 Sep 10;233(5):e1215–25. doi: 10.1093/infdis/jiaf474 (PMC13175607; doi:10.1093/infdis/jiaf474)
Supplement: jiaf474_Supplementary_Data [file jiaf474_supplementary_data.zip › Supplemental_Table1.docx]

**Supplemental Table 1. nCounter code set used for transcriptional analysis**

| Accession number | Name | Description | Function | Transcripts detected |
| --- | --- | --- | --- | --- |
| IT4_var19.1 | var19 | Plasmodium falciparum erythrocyte membrane protein 1 (PfEMP1) | Cytoadhesion | Yes (IT4var19 only) |
| PfIT_120006100 | FCR3CSA | PfEMP1 | Cytoadhesion | Yes (FCR3CSA only) |
| PF3D7_0201900 | PfEMP3 | Plasmodium falciparum erythrocyte membrane protein 3 (PfEMP3) | Cytoadhesion and erythrocyte cytoskeleton modification | Yes |
| PF3D7_0202200 | PTP1 | PfEMP1 trafficking protein | PfEMP1 trafficking and cytoadherence | Yes |
| PF3D7_0731100 | PTP2 (PHISTc) | PfEMP1 trafficking protein | PfEMP1 trafficking and cytoadherence | Yes |
| PF3D7_1478600 | PTP3 | PfEMP1 trafficking protein | PfEMP1 trafficking and cytoadherence | Yes |
| PF3D7_0730900 | PTP4 | PfEMP1 trafficking protein | PfEMP1 trafficking and cytoadherence | Yes |
| PF3D7_1002100 | PTP5 | PfEMP1 trafficking protein | PfEMP1 trafficking, cytoadhesion, and IE rigidity | Yes |
| PF3D7_1302000 | PTP6 | PfEMP1 trafficking protein | PfEMP1 trafficking and cytoadherence | Yes |
| PF3D7_0102600 | FIKK1 | serine/threonine protein kinase, FIKK family | Phosphorylation | Yes |
| PF3D7_0424500 | FIKK4.1 | serine/threonine protein kinase, FIKK family | Phosphorylation, modulate VAR2CSA PfEMP1 surface display | Yes |
| PF3D7_0424700 | FIKK4.2 | serine/threonine protein kinase, FIKK family | Phosphorylation | Yes |
| PF3D7_1016400 | FIKK10.1 | serine/threonine protein kinase, FIKK family | Phosphorylation, associates with Maurer's clefts | Yes |
| PF3D7_1039000 | FIKK10.2 | serine/threonine protein kinase, FIKK family | Phosphorylation | Yes |
| PF3D7_1149300 | FIKK11 | serine/threonine protein kinase, FIKK family | Phosphorylation | No |
| PF3D7_0902300 | FIKK9.4 | serine/threonine protein kinase, FIKK family | Phosphorylation, associates with Maurer's clefts | No |
| PF3D7_0202000 | KAHRP | Knob associated histidine rich protein | Knob assembly | Yes |
| PF3D7_0201800 | KAHSP40 | Knob associated heat shock protein 40 | Knob assembly | Yes |
| PF3D7_0532400 | lyMP-PHISTb | Lysine-rich membrane associated PHISTb protein | Knob assembly | Yes |
| PF3D7_1039100 | PF10_0381 | Dnaj protein, putative, pseudogene | Knob formation | No |
| PF3D7_0935900 | REX1 | Ring exported protein 1 | Shape and organization of Maurer's clefts, PfEMP1 trafficking | Yes |
| PF3D7_0113900 | GEXP10 | Gametocyte exported protein 10/Plasmodium exported protein (hyp8) | Maurer's cleft protein | Yes |
| PF3D7_1301700 | GEXP-07 | Gametocyte exported protein | Maurer's cleft protein | Yes |
| PF3D7_1149000 | PF332 | Antigen 332, DBL-like protein | Maurer's cleft protein, IE rigidity | Yes |
| PF3D7_1370300 | MAHRP1 | Membrane associated histidine-rich protein | Maurer's cleft protein, PFMEP1 trafficking | Yes |
| PF3D7_0501300 | SBP1 | Skeleton binding protein 1 | Maurer's cleft protein, involved in PfEMP1 trafficking | Yes |
| PF3D7_0702400 | SEMP1 | Small exported membrane protein 1 | Associates with Maurer's clefts | Yes |
| PF3D7_1301400 | HYP12 | Plasmodium exported protein (hyp12) | Unknown function, associates with Maurer's cleft | Yes |
| PF3D7_0936000 | REX2 | Ring exported protein 2 | Associates with Maurer's clefts | No |
| PF3D7_0324100 | Pfmc-2TM | Maurer's cleft two transmembrane protein | Maurer's cleft protein | No |
| PF3D7_0201600 | RLP1 | PHISTb domain-RESA-like protein1 (RLP1) | Chaperone for cytoadhesion machinery | Yes |
| PF3D7_0113700 | HSP40 | Heat shock protein 40 | co-chaperone | Yes |
| PF3D7_0220100 | DnaJ | DnaJ protein, putative | Chaperone | Yes |
| PF3D7_0501100 | JDP | J domain protein, heat shock protein 40, type II | co-chaperone, J-dots | Yes |
| PF3D7_0831700 | HSP70x | Exported heat shock protein 70 | Chaperone, J-dots | Yes |
| PF3D7_0102200 | RESA | Ring-infected erythrocyte surface antigen | Erythrocyte cytoskeleton | Yes (It4var19 only) |
| PF3D7_0919800 | TLD protein | TLD domain-containing protein | Putative binding to host cell cytoskeleton containing domain | No |
| PF3D7_0424600 | PHISTb | Plasmodium exported protein (PHISTb), Plasmodium helical interspersed subtelomeric protein | Unknown, presumed host cell remodeling | Yes |
| PF3D7_0501200 | PIESP2 | Parasite exported protein and infected erythrocyte surface protein | Virulence | Yes |
| PF3D7_0601000 | PfEPF4 family | Plasmodium falciparum exported protein family 4 | Merozoite release from erythrocytes | Yes |
| PF3D7_1016300 | GBP | Glycophorin binding protein 130 | Merozoite invasion | Yes |
| PF3D7_1101800 | PfEPF1 family | Plasmodium falciparum exported protein family 1 | Unknown | Yes |
| PF3D7_1133700 | FHA Protein | FHA domain containing Protein, putative | Unknown | Yes |
| PF3D7_1401000 | GBP homolog | Glycophorin binding protein homolog | Unknown | No |
| Non-exported |  |  |  |  |
| PF3D7_1328800 | PfSir2a | Transcriptional regulatory protein sir2a | Plays a role in silencing of var genes | No |
| PF3D7_1451400 | PfSir2b | Transcriptional regulatory protein sir2b | Plays a role in silencing of var genes | Yes |
| PF3D7_1323500 | Plasmepsin V | Plasmepsin V | Protease essential for protein export in P. falciparum | Yes |
| PF3D7_0206700 | ALS | Adenylosuccinate lyase | Housekeeping | Yes |
| PF3D7_0717700 | STS | Seryl-tRNA synthetase | Housekeeping | Yes |
| PF3D7_1462800 | GAPDH | Glyceraldehyde 3-phosphate dehydrogenase | Housekeeping | Yes |
